# Effects of waterborne cadmium exposure on hematological parameters, oxidative stress, and stress-related genes in crucian carp (*Carassius auratus*)

**DOI:** 10.7717/peerj.21528

**Published:** 2026-07-08

**Authors:** Tianhui Gao, Yuanhua Xu, Jian Song, Min Lou, Chunhao Shen, Jianli Xiong

**Affiliations:** 1 School of Life Sciences (School of Ecological Forestry), Mianyang Normal University, Mianyang, Sichuan, China; 2 Forest Ecology and Conservation in the Upper Reaches of the Yangtze River Key Laboratory of Sichuan Province, Mianyang Normal University, Mianyang, Sichuan, China; 3 Key Laboratory of Research and Conservation of Biological Diversity in Minshan Mountain of National Park of Giant Pandas at Mianyang Normal University of Sichuan Provincial Department of Education, Mianyang, Sichuan, China; 4 School of Geography and Environment, Mianyang Normal University, Mianyang, Sichuan, China

**Keywords:** Aquatic environment, Heavy metal pollutant, Hematology, Antioxidant response, Gene expression

## Abstract

Cadmium (Cd) is a non-essential and highly toxic heavy metal widely present in aquatic environments. However, studies investigating the toxic effects of Cd on crucian carp (*Carassius auratus*) are relatively few. This study aimed to evaluate the toxic effects of Cd by assessing hematological parameters, antioxidant responses, and the expression of stress-related genes in *C. auratus* exposed to waterborne Cd. A total of 180 healthy *C. auratus* (19.43 ± 1.5 cm and 170.00 ± 3.04 g) were exposed to Cd at concentrations of 0, 2, and 4 mg/L for 8 weeks, with three replicates per treatment. The exposure to Cd resulted in significant reductions in red blood cell (RBC) count, hemoglobin (Hb) concentration, and hematocrit (Hct) values. Moreover, the levels of total superoxide dismutase (T-SOD), catalase (CAT), and glutathione peroxidase (GPx) in the liver also decreased significantly, whereas malondialdehyde (MDA) content was increased significantly. The expression levels of *CAT*, copper (Cu)/zinc (Zn)-superoxide dismutase (*Cu/Zn-SOD*), heat shock protein 70 (*HSP70*), heat shock protein 90 (*HSP90*), and metallothionein (*MT2)* genes were significantly upregulated. Overall, Cd exposure adversely affected the hematological parameters, induced oxidative stress, and altered the expression levels of stress-related genes. This study not only provides new insights into the toxic effects of Cd in *C. auratus* but also contributes to environmental monitoring research.

## Introduction

Cadmium (Cd) is a silver-white, nonessential transition trace element with no known physiological function. It primarily occurs naturally in the sulfide ores of zinc, lead, and copper. Cd has been recognized as one of the 10 most toxic metals in the Priority List of Hazardous Substances (ATSDR 2007) (Wang et al., 2019) because of its environmental toxicity, strong tendency for bioaccumulation, and poor degradability (Liu et al., 2022; Genchi et al., 2020). However, it has a wide range of adverse effects on multiple organs, including the liver, kidney, stomach, brain, breast, lung, nasopharynx, pancreas, prostate, ovary, and urinary bladder (Liu et al., 2022; Genchi et al., 2020). Furthermore, Cd accumulates in the body and exerts its toxic effects through the food chain (Satarug, 2018). Cd persists in plants and animals with a long biological half-life of approximately 25–30 years (Genchi et al., 2020). Although natural Cd concentrations are extremely low (Genchi et al., 2020; Cheng et al., 2022), human activities, such as fossil fuel combustion, mining, dye and plastics manufacturing, phosphate fertilizer applications, electroplating, and sewage sludge disposal (Genchi et al., 2020; Gómez-Mendikute & Cajaraville, 2003; Hua et al., 2019; Le Croizier et al., 2018; Othmani et al., 2022), have significantly increased environmental Cd levels. Consequently, Cd pollution has become a serious environmental concern globally.

Cd is ubiquitous in aquatic environments, where it exists as ions or compounds bound to inorganic anions and organic ligands (Ahmadniaye, Javadmanesh & Safari, 2020). As a result, Cd has become a persistent contaminant in aquatic ecosystems (Sun et al., 2020; Burger & Gochfeld, 2009. As vital components of aquatic food webs, fish are particularly sensitive to Cd exposure (Liu et al., 2022; Paul & Small, 2021). Cd enters their body through respiration and digestion (Luo et al., 2020; Othmani et al., 2022; Ahmadniaye, Javadmanesh & Safari, 2020), subsequently distributing to and accumulating in various target organs, including the liver, kidney, gills, intestine, and integumentary system. This bioaccumulation induces multisystem toxicity and compromises structural integrity and physiological functions across multiple organ systems, impairing antioxidant defense, reproductive regulation, immune responses, and neural functions (Liu et al., 2022; Mielcarek et al., 2022). The underlying mechanism may be that Cd exposure promotes the generation of reactive oxygen species (ROS), resulting in oxidative damage to various molecules, DNA damage, mitochondrial membrane depolarization, mtDNA mutations, and apoptosis (Genchi et al., 2020; Lee et al., 2023; Choi et al., 2007). Owing to the toxic effects of Cd on fish, China’s Fishery Water Quality Standard stipulates that the allowable concentration of Cd should not exceed 0.005 mg/L.

Crucian carp (*Carassius auratus*) is a widely cultured freshwater fish in China because of its good meat quality, high nutritional value, strong disease resistance, high reproductive activity, and strong survivability (Hu et al., 2023; Liu et al., 2022). It is used as a biological model because it is easy to grow and can readily adapt to laboratory settings (Ahmadniaye, Javadmanesh & Safari, 2020). The toxic effects of Cd on *C. auratus* remain unclear. To date, only a few studies have investigated the toxic effects of Cd on gill and liver superoxide dismutase (SOD) (Yang et al., 2003) and liver catalase (CAT) activity (Xu, Xiong & Wang, 2006; Hu, Duan & Tang, 2011). The transport, distribution, and elimination of Cd are mediated primarily through the circulatory system (Li et al., 2020). Thus, the hematological parameters, such as the red blood cells (RBC) count, hemoglobin (Hb) content, and the hematocrit (Hct) value (Kim & Kang, 2017; Yu et al., 2023; Won et al., 2023), are highly sensitive to Cd (Lee, Choi & Kim, 2022). Among fish organs, the liver serves as the primary detoxification organ and essential intermediate metabolic organ (Zhao et al., 2024). Hence, long-term Cd exposure can lead to its accumulation in the liver (Liu et al., 2022; Das, Kar & Patra, 2023). Furthermore, fish liver is particularly susceptible to Cd toxicity, likely because of its ability to synthesize metallothionein, a Cd-induced protein that sequesters toxic Cd ions to exert cytoprotective effects (Liu et al., 2022). This study was conducted to analyze the effects of waterborne Cd exposure on hematological parameters, antioxidant response and the expression of stress-related gene in the liver of *C. auratus*. We hypothesized that Cd exposure would affect the hematological parameters, induce oxidative stress, and alter the expression levels of stress-related genes. To test this hypothesis, we evaluated the effects of waterborne Cd at concentrations of 0, 2, and 4 mg/L on RBC count, Hb concentration, and the Hct value. We also measured total superoxide dismutase (T-SOD), catalase (CAT), and glutathione peroxidase (GPx) activities and malondialdehyde (MDA) content, as well as the expression levels of *CAT*, copper (Cu)/zinc (Zn)-superoxide dismutase (*Cu/Zn-SOD*), heat shock protein 70 (*HSP70*), heat shock protein 90 (*HSP90*), and metallothionein (*MT2)* in the liver.

## Materials and Methods

### Experimental animals and care

A total of 180 healthy *C. auratus* fish, with an average body weight of (170.00 ± 3.04) g and body length of (19.43 ± 1.5) cm, were purchased from a local fish breeding base in Mianyang, Sichuan, China. Fish with good clinical health and vigor were selected. These fish were randomly assigned to nine tanks (55 cm × 40 cm × 40 cm) containing 24 L of aerated and filtered dechlorinated water, with 20 fish in each tank. Before starting the experiment, the fish were acclimated for 7 days under conditions simulating the experimental environment: water temperature 26.0 °C ± 1.0 °C, dissolved oxygen ≥ 6.0 mg/L, ammonia <0.5 mg/L, nitrites <0.05 mg/L, pH 7.0 ± 0.2, and a photoperiod of 12-h light: 12-h dark. The fish were fed a basal diet twice daily (9:00 a.m. and 6:00 p.m.) at 2% of their body weight. Uneaten feed was collected after 30-min feeding. Water in each tank was exchanged twice daily by replacing one-third of the total volume. While changing water, a siphon hose was used to draw water from the bottom to remove settled metabolic waste, thereby keeping the water clean.

### Experimental reagents

CdCl_2_ ⋅ 2.5H_2_O (purity >99%) used in this study was purchased from FuChen Chemical Reagent Co., Ltd. (Tianjin, China). A stock solution with a concentration of one g/L was prepared by dissolving 2.0316 g of CdCl_2_ ⋅ 2.5H_2_O in one L of distilled water and stored at 4 °C. The working solutions were prepared by diluting the stock solution to the desired concentrations before use.

### Experimental design

After acclimation, the fish were exposed to waterborne Cd. The study found that the median lethal concentration (LC_50_) of Cd for 150.0 g *C. auratus* over a 96-h period was 15.28 mg/L, and the safe concentration was 1.528 mg/L (Wei et al., 2022). In this experiment, the weight of *C. auratus* averaged around 170.0 g; the concentration gradient setting was based on the results of Wei et al. (2022). The experiment design comprised three groups: control group (CK, 0 mg/L), experimental group 1 (T1, Cd concentration two mg/L, 1.31 times the safe concentration), and the experimental group 2 (T2, Cd concentration four mg/L, 2.62 times the safe concentration), each with three replicates. The exposure lasted for 8 weeks. The experimental conditions were identical to those used during acclimation. After each water exchange, the Cd solution was replenished to maintain the designated concentrations. After 8 weeks of Cd exposure, fish were randomly selected from each group and anesthetized with 300 mg/L of tricaine methane sulfonate-222 (MS-222). The blood samples were collected from the caudal vein using a 1-mL syringe without anticoagulant, transferred into a 2-mL sterile enzyme-free Eppendorf tube, and placed in a refrigerator at 4 °C for 30 min. Then, the fish were aseptically dissected on ice. The liver was excised, placed in a cryotube, immediately frozen in liquid nitrogen, and subsequently stored at −80 °C until use for analyzing the antioxidant response and gene expression.

### Analysis of hematological parameters

A total of 16 fish were selected from each group for blood sample collection, and their hematological parameters were analyzed immediately after collection following the method proposed by Lin, Bai & Xiong (2022). RBC count was determined manually using a Neubauer hemocytometer under an Olympus CX31 light microscope (Olympus, Tokyo, Japan). Hb concentration was measured using a Sahli hemoglobinometer (MC, Jiangyan Huihong Experimental Instrument Factory, Taizhou, Jiangsu Province, China). Hct was calculated from the proportion of the blood cell volume in the total blood volume after centrifuging (Xiangzhi Centrifuge TG12; Changsha Xiangzhi Centrifuge Instrument Co Ltd, relative centrifugation force: 14,800 g) the blood samples in microhematocrit tubes at 12,000 rpm for 5 min.

### Analysis of atioxidant response

A total of eight fish were selected from each group for analyzing the antioxidant response. The antioxidant response of the liver was quantified using commercial assay kits following the manufacturer’s protocols. The CAT assay kits (G4307) were purchased from Wuhan Servicebio Technology Co., Ltd., Wuhan, Hubei, China, and the T-SOD (A001), GPx (A005), and MDA (A003) assay kits were purchased from Nanjing Jiancheng Bioengineering Institute.

### Quantitative polymerase chain reaction analysis

A total of six fish were selected from each group for quantitative polymerase chain reaction (qPCR) analysis. Total RNA was extracted from liver tissues using a TRIzol kit (Wuhan Servicebio Technology CO., LTD, Wuhan, China, G3013) following the manufacturer’s protocol. RNA quality was determined by 1% agarose gel electrophoresis, whereas RNA quantity was measured using a NanoDrop 2000 spectrophotometer (Thermo Scientific, Waltham, MA, USA). Samples with A260/A280 ratios between 1.8 and 2.0 were used for cDNA synthesis. Reverse transcription was performed using the reverse transcription kit containing SweScript All-in-One RT SuperMix for qPCR (one-step gDNA remover) (Wuhan Servicebio Technology CO., LTD, Wuhan, China, G3337). The reaction volume was 20 µL, containing 4 µL of 5× SweScript All-in-One SuperMix, 1 µL of gDNA remover, and 10 µL of total RNA. The reaction program was as follows: 25 °C for 5 min, followed by 42 °C for 30 min and 85 °C for 5 s. Real-time quantitative polymerase chain reaction (RT-qPCR) was performed using the LightCycler^®^ 480 System (Roche, Basel, Switzerland) with the FastStar Universal SYBR Green Master (Roche, Basel, Switzerland). The reaction volume was 15 µL, containing 7.5 µL of SYBR Green Master Mix, 1.5 µL of each primer, and 2.0 µL of cDNA. The PCR reaction program was as follows: 95 °C for 30 s, followed by 40 cycles of 95 °C for 15 s and 60 °C for 30 s. All qRT-PCR analyses were performed with three biological replicates. The relative gene expression of the target genes was calculated using the 2^−ΔΔCt^ method (Livak & Schmittgen, 2001). The primers to detect genes, including *CAT*, *Cu/Zn-SOD*, *HSP70*, *HSP90*, and *MT2*, arelisted in Table 1, and glyceraldehyde-3-phosphate dehydrogenase (*GAPDH*) was used as the internal reference.

**Table 1:** Primers used in this study.

**Primer name**	**primer sequence (5′to 3′)**	**Product size (bp)**	**GenBank accession number**
*GAPDH*	F: AGGCATTCTGGGATACACGGAG	242	XM_026284269.1
	R: GATGGGAGAACGGTGGGTCA		
*CAT*	F: AATACTGTTGGGTGGCGGTAAT	266	XM_026238665.1
	R: GACCCGCTGTCATTGAGTTT		
*Cu/Zn-SOD*	F: CAACCCTCATAATCAAACTCACG	257	XM_026273656.1
	R: CTATAACACCACAGGCCAGACG		
*HSP70*	F: AACCGCAATTAAGCCTGACAAA	71	XM_026208615.1
	R: CCAGGTCAATCCCAATAGCAAC		
*HSP90*	F: CCAAACACAACGATGACGAGCA	179	DQ872650.1
	R: GGAGTGTTTCTTGACCACTTCCTTC		
*MT2*	F: AAGACTGGAGCTTGCAACTGTG	142	XM_026230631.1
	R: CGCAGGAATTGCCCTTACAC		

**Notes.**

CAT catalase g Cu/Zn-SOD Cu/Zn superoxide dismutase HSP70 heat shock protein 70 HSP90 heat shock protein 90 MT2 metallothionein 2 F forward R reverse

### Statistical analysis

The data were analyzed using SPSS 23.0 (SPSS Inc., Armonk, NY, USA) and expressed as mean ± standard error (mean ±  S.E.). The graphs were generated using GraphPad Prism 5.01 (GraphPad Software Inc., La Jolla, CA, USA). The groups were compared using Welch’s ANOVA followed by the Games–Howell test. A *p*- value less than 0.05 indicated a significant difference.

### Ethics statement

All experiments were carried out according to protocols approved by the Institutional Animal Care and Use Committee (IACUC) of School of Life Sciences (School of Ecological Forestry), Mianyang Normal University (202304001).

## Results

### Hematological parameters

**Figure 1:**
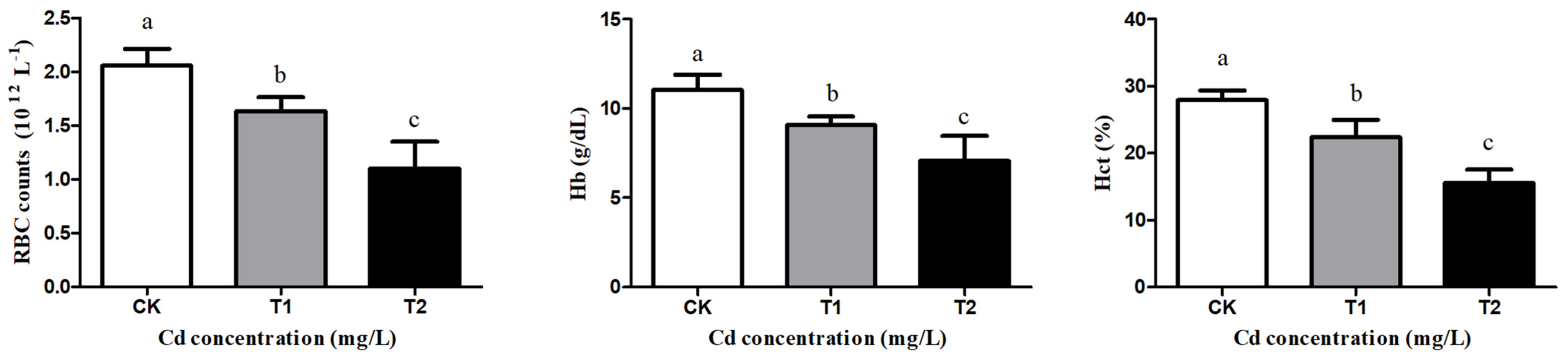
Hematological parameters of crucian carp exposed to waterborne Cd. Values were expressed as mean ± S.E (*n* = 16). Bars with different letters are significantly different (*P* < 0.05).

The hematological parameters of *C. auratus* exposed to waterborne Cd are shown in Fig. 1. Cd exposure significantly decreased the RBC counts, Hb concentrations, and Hct values compared with those in control group (*p* < 0.001). Moreover, the hematological parameters decreased significantly (*p* < 0.001) with increase in Cd concentration.

### Antioxidant responses

**Figure 2:**
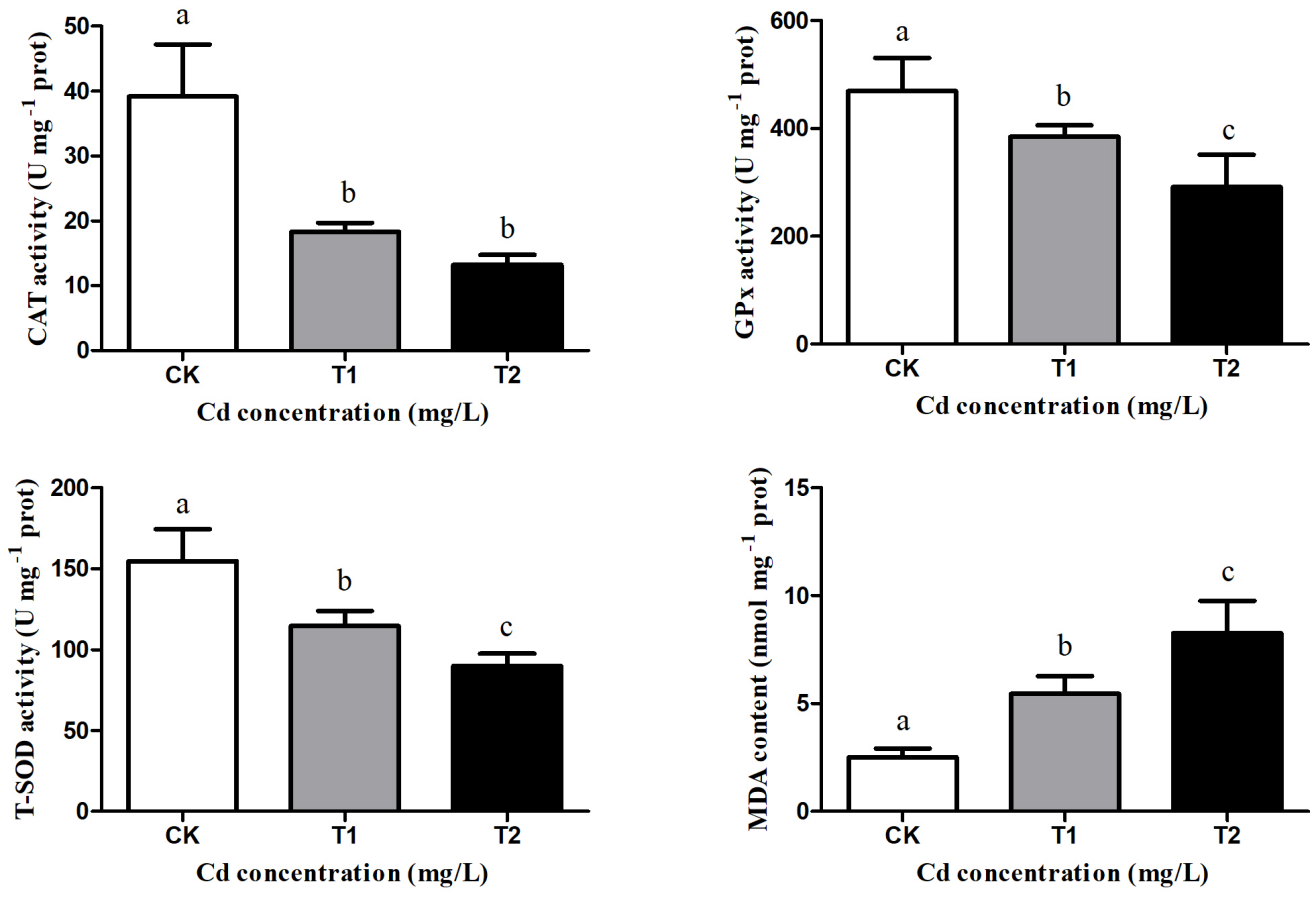
Antioxidant capability in the liver of crucian carp exposed to waterborne Cd. Data are expressed as the mean ± S.E. (*n* = 8). Bars with different letters are significantly different (*p* < 0.05).

The antioxidant responses in the liver of *C. auratus* exposed to waterborne Cd are presented in Fig. 2. Cd exposure significantly decreased CAT, GPx, and T-SOD activities (*P* < 0.05) and increased MDA content (*P* <  0.05) compared with those in control group. CAT activity was not significantly different (*p* > 0.05) between the two Cd-exposed groups (two mg/L *vs* four mg/L), whereas GPx and T-SOD activities decreased significantly (*p* <  0.05). Moreover, MDA content increased (*p* < 0.05) with an increase in Cd concentration.

### Gene expression

**Figure 3:**
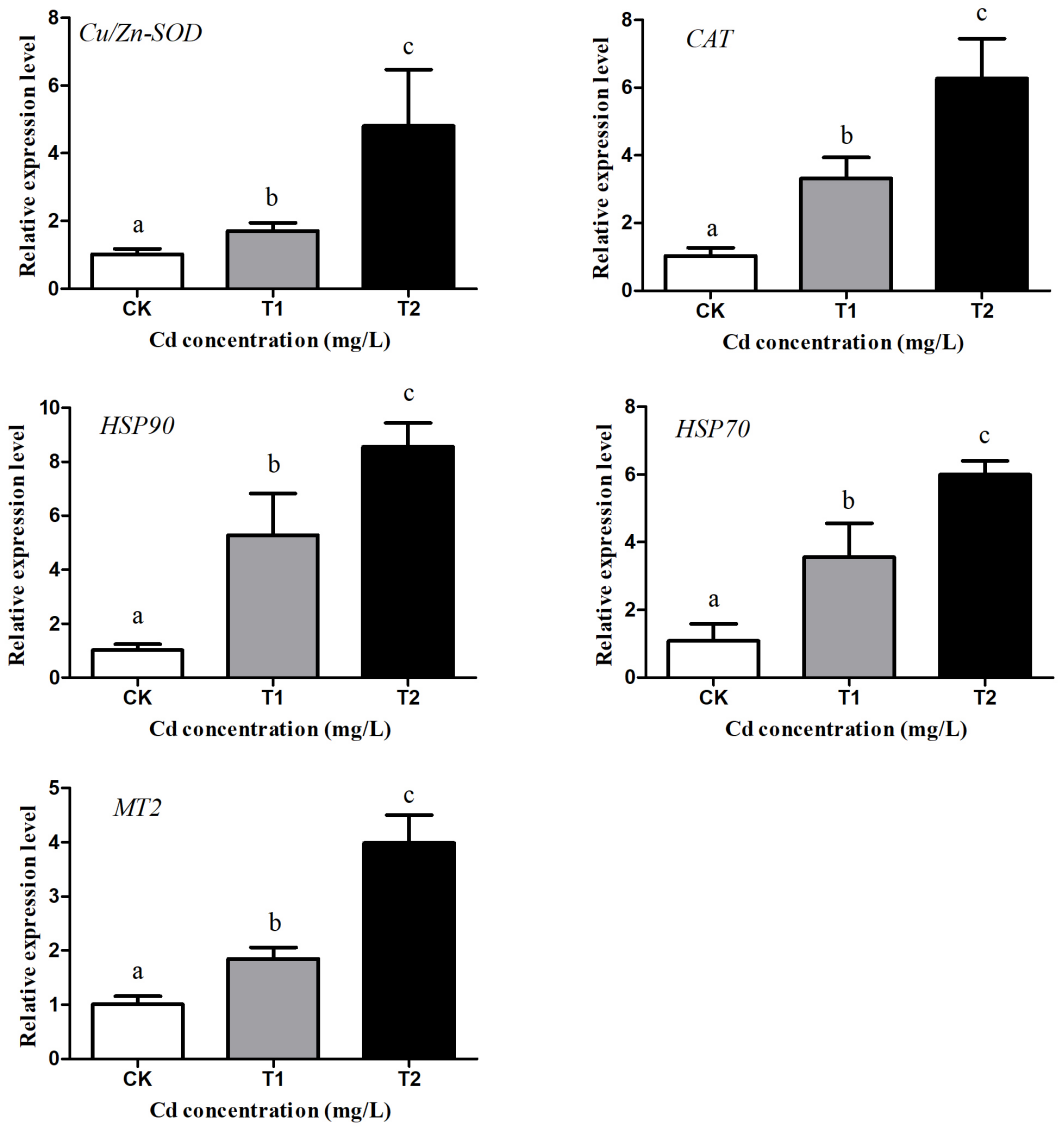
Expression of genes in the liver of crucian carp exposed to waterborne Cd. Data are expressed as the mean ± S.E. (*n* = 6). Bars with different letters are significantly different (*p* < 0.05).

The expression levels of the genes (*CAT*, *Cu/Zn-SOD*, *HSP70*, *HSP90*, and *MT2*) in the liver of *C. auratus* exposed to waterborne Cd are presented in Fig. 3. Cd exposure significantly increased the expression levels of the target genes compared with those in control group (*p* < 0.05). Furthermore, the expression levels of the target genes decreased significantly (*p* < 0.05) with an increase in Cd concentration.

## Discussion

This study explored the effects of waterborne Cd exposure on hematological parameters, liver antioxidant responses, and stress-related gene expression in *C. auratus*. The results showed that Cd exposure significantly decreased the hematological parameters, and the activities of CAT, GPx, and T-SOD in the liver, but increased liver MDA content and the expression of stress-related genes (*CAT*, *Cu/Zn-SOD*, *HSP70*, *HSP90*, and *MT2*). These results support our hypothesis that Cd exposure affects the hematological parameters, induce oxidative stress, and alters the expression levels of stress-related genes.

Hematological parameters are a valuable index to examine health status, as well as physiological and pathological changes (Ahmed, Reshi & Fazio, 2020; Fazio, 2019; Salaah et al., 2025) because they reflect the physiological condition and health state of fish (Yu et al., 2023). Both intrinsic and extrinsic factors can cause a huge variability in the hematological parameters in fish (Ahmed, Reshi & Fazio, 2020). Also, hematological parameters are highly impacted by Cd toxicity (Won et al., 2023). Many studies have documented significant reductions in the hematological parameters of fish exposed to Cd-contaminated environments. For example, Hct value and Hb concentration in juvenile *Paralichthys olivaceus* (Lee, Choi & Kim, 2022), *Mystus seenghala* (Fazio et al., 2022), and RBCs, Hb, and Hct in *Oreochromis niloticus* (Al-Asgah et al., 2015), *Catla catla* (Naz et al., 2021), and *Platichthys stellatus* (Won et al., 2023) decreased significantly after Cd exposure. Consistent with previous findings, the present study showed that RBC count, Hb concentration, and Hct value decreased significantly in *C. auratus* exposed to Cd, with a dose-dependent effect. This might be attributed to various toxic effects of Cd on the circulatory system of fish, including impaired erythropoiesis, reduced life span, and disequilibrium of osmotic pressure in RBCs (Won et al., 2023).

Oxidative stress refers to a metabolic imbalance caused by excessive ROS production and insufficient antioxidant defense (Justi et al., 2025; Oršolić & Jembrek, 2025). ROS, such as superoxide anion radicals (O_2_^•^−^^), hydrogen peroxide (H_2_O_2_), hydroxyl radicals (•HO), and singlet oxygen (^1^O_2_), are generated during mitochondrial oxidative metabolism and cellular responses to xenobiotics, cytokines, and bacterial invasion (Ray, Huang & Tsuji, 2012). Cd exposure increases ROS production (Genchi et al., 2020). Excessive ROS accumulation causes structural damage to biological macromolecules, disrupts DNA replication and repair, induces mitochondrial membrane depolarization (Genchi et al., 2020; Zhang, 2018), and promotes MDA production (Liu et al., 2022). The antioxidant defense system in fish neutralizes ROS through antioxidant enzymes (Perra & Manca, 2025), such as T-SOD, CAT, and GPx. T-SOD scavenges excess free radicals, thus preventing cellular damage (Li et al., 2008); GPx reduces hydrogen peroxide and organic peroxides to their corresponding alcohols (Pei et al., 2023); and CAT decomposes hydrogen peroxide into water and oxygen (Das et al., 2019). MDA is the main oxidation product of lipid peroxidation, and its concentration is often used to measure the extent of oxidative damage (Liu et al., 2008). Previous studies have demonstrated that Cd exposure reduces the activity of antioxidant enzymes and increases MDA content in fish. For example, Cd exposure decreased SOD and CAT activities and significantly increased MDA content in the liver of *C. auratus gibelio* (Wang et al., 2020) and the gill of *Cyprinus carpio L.* (Chen et al., 2021). Consistent with these findings, the present study demonstrated that Cd exposure significantly decreased T-SOD, CAT, and GPx activities and significantly increased MDA content in the liver of *C. auratus*. Moreover, except for CAT, with no significant difference in activity between the two exposure groups, all other measured indicators exhibited dose-dependent increases or decreases on exposure to elevated Cd concentrations. This might be attributed to the efficient removal of H_2_O_2_ by GPx, in which case H_2_O_2_ concentration was too low to effectively activate CAT. These results indicated that Cd exposure caused marked oxidative stress in *C. auratus*, leading to increased ROS production and reduced antioxidant capacity of the antioxidant system.

Antioxidant enzyme genes in fish, such as *CAT* and *SOD*, as well as oxidative stress–related genes, such as metallothionein (*MT*) and heat shock proteins (*HSPs*), are important components of the stress defense mechanism (Liu et al., 2022). *Cu/Zn-SOD* and *CAT* are antioxidant enzyme genes playing crucial roles in the overall defense mechanism and strategy of biological systems (Ighodaro & Akinloye, 2018). *HSPs* are stress-regulating proteins highly expressed in response to environmental factors, such as temperature, heavy metals, reactive oxygen, and salinity. These proteins play important roles in protecting cells from toxic trauma, and are often used as a biochemical marker of exposure to metal toxicity (Li et al., 2019). *MTs* are cysteine-rich non-enzymatic antioxidants that bind to metal ions, reducing free-radical production and protecting cells from the toxic effects of heavy metals (Sun et al., 2024). Cd exposure impacts the expression of antioxidant enzymes and oxidative stress-related genes. For example, Cd exposure significantly increased the expression of *Cu/Zn-SOD*, and *CAT* genes in the liver and spleen of *Takifugu obscurus* (Kim et al. , 2010), *HSP47*, *HSP60*, *HSP70*, *HSP90*, and *MT2* genes in the liver of *Cyprinus carpio* (Jiang et al., 2016) and *Labeo rohita* (Giri et al., 2016), *HSP70* gene in the liver of *P. olivaceus* (Lee, Choi & Kim, 2022), and *HSP70* and *HSP90* genes in the liver of common carp (Wang et al., 2022). In the present study, *Cu/Zn-SOD*, *CAT*, *HSP70*, *HSP90*, and *MT2* gene expression levels in the liver of *C. auratus* increased significantly in response to the oxidative stress caused by Cd exposure.

The toxic effects of Cd on fish are primarily related to Cd accumulation within their bodies. This accumulation is influenced by various factors, including Cd concentration in the environment, duration of exposure, specific organ type, as well as feeding habits and routes (Al-Asgah et al., 2015). In this study, the concentration range of Cd was limited, and the measurement data regarding Cd accumulation in tissues and the actual Cd concentrations in water bodies were lacking. These limitations prevented a comprehensive and in-depth examination of the toxic effects of Cd on *C. auratus*. Therefore, further studies should optimize the concentration gradient and involve more systematic and comprehensive investigations to elucidate the toxicological mechanisms of Cd in *C. auratus*, thereby providing a robust scientific basis for the ecological risk assessment of Cd in aquatic ecosystems.

## Conclusion

This study evaluated the effects of waterborne Cd exposure on hematological parameters, oxidative stress, and stress-related gene expression in *C. auratus*. The results indicated that Cd exposure induced oxidative stress and exerted adverse effects on hematological parameters, antioxidant responses, and the expression of stress-related genes in *C. auratus*. Stress-related genes may serve as robust biomarkers for the early detection of environmental pollution. Hence, the present study not only provides new insights into the toxic effects of Cd in *C. auratus* but also offers valuable methodological support for environmental monitoring research.

##  Supplemental Information

10.7717/peerj.21528/supp-1 Supplemental Information 1 Graphical abstract Cadmium exposure adversely impacts hematological parameters, induces oxidative stress, and results in significant alterations in the expression levels of stress-related genes.

10.7717/peerj.21528/supp-2 Supplemental Information 2 ARRIVE checklist

10.7717/peerj.21528/supp-3 Supplemental Information 3 MIQE checklist

10.7717/peerj.21528/supp-4 Supplemental Information 4 Hematological parameters

10.7717/peerj.21528/supp-5 Supplemental Information 5 Antioxidant data

10.7717/peerj.21528/supp-6 Supplemental Information 6 Genes data

## Additional Information and Declarations

### Data Availability

The following information was supplied regarding data availability: The data is available in the Supplemental Files.

## References

[ref-1] Ahmadniaye MH, Javadmanesh A, Safari O (2020). Improvement of non-specific immunity, growth, and activity of digestive enzymes in *Carassius auratus* as a result of apple cider vinegar administration to diet. Fish Physiology and Biochemistry.

[ref-2] Ahmed I, Reshi QM, Fazio F (2020). The influence of the endogenous and exogenous factors on hematological parameters in different fish species: a review. Aquaculture International.

[ref-3] Al-Asgah NA, Abdel-Warith AA, Younis EM, Allam HY (2015). Haematological and biochemical parameters and tissue accumulations of cadmium in *Oreochromis niloticus* exposed to various concentrations of cadmium chloride. Saudi Journal of Biological Sciences.

[ref-4] Burger J, Gochfeld M (2009). Comparison of arsenic, cadmium, chromium, lead, manganese, mercury and selenium in feathers in bald eagle (*Haliaeetus leucocephalus*), and comparison with common eider (*Somateria mollissima*), glaucous-winged gull (*Larus glaucescens*), pigeon guillemot (*Cepphus columba*), and tufted puffin (*Fratercula cirrhata*) from the Aleutian Chain of Alaska. Environmental Monitoring and Assessment.

[ref-5] Chen J, Chen D, Li J, Liu Y, Gu X, Teng X (2021). Cadmium-induced oxidative stress and immunosuppression mediated mitochondrial apoptosis via JNK-FoxO3a-PUMA pathway in Common Carp *(Cyprinus carpio* L.) gills. Aquatic Toxicology.

[ref-6] Cheng C, Ma H, Liu G, Fan S, Guo Z (2022). Mechanism of cadmium exposure induced hepatotoxicity in the mud crab (*Scylla paramamosain*): activation of oxidative stress and Nrf2 signaling pathway. Antioxidants.

[ref-7] Choi CY, An KW, Nelson ER, Habibi HR (2007). Cadmium affects the expression of metallothionein (MT) and glutathione peroxidase (GPX) mRNA in goldfish, *Carassius auratus*. Comparative Biochemistry and Physiology. Toxicology & pharmacology.

[ref-8] Das N, Jana CK, Yan L, Nandi A, Signorini C (2019). Role of catalase in oxidative stress- and age-associated degenerative diseases. Oxidative Medicine and Cellular Longevity.

[ref-9] Das S, Kar I, Patra AK (2023). Cadmium induced bioaccumulation, histopathology, gene regulation in fish and its amelioration –a review. Journal of Trace Elements in Medicine and Biology.

[ref-10] Fazio F (2019). Fish hematology analysis as an important tool of aquaculture: a review. Aquaculture.

[ref-11] Fazio F, Habib SS, Naz S, Hashmi MAH, Saoca C, Ullah M (2022). Cadmium sub-lethal concentration effect on growth, haematological and biochemical parameters of *Mystus seenghala* (Sykes, 1839). Biological Trace Element Research.

[ref-12] Genchi G, Sinicropi M, Lauria G, Carocci A, Catalano A (2020). The effects of cadmium toxicity. International Journal of Environmental Research and Public Health.

[ref-13] Giri SS, Sen SS, Jun JW, Sukumaran V, Park SC (2016). Immunotoxicological effects of cadmium on *Labeo rohita*, with emphasis on the expression of HSP genes. Fish & Shellfish Immunology.

[ref-14] Gómez-Mendikute A, Cajaraville MP (2003). Comparative effects of cadmium, copper, paraquat and benzo[a]pyrene on the actin cytoskeleton and production of reactive oxygen species (ROS) in mussel haemocytes. Toxicology in Vitro.

[ref-15] Hu X, Bai J, Liu R, Lv A (2023). Comprehensive transcriptomics and proteomics analysis of *Carassius auratus* gills in response to aeromonas hydrophila. Fish and Shellfish Immunology Reports.

[ref-16] Hu R, Duan H, Tang Z (2011). The effect of Cadmium an Plumbum on activities of catalase from hepatopancreas of *Carassius auratus*. Life Science Research.

[ref-17] Hua X, Huang X, Tian J, Dong D, Liang D, Guo Z (2019). Migration and distribution of cadmium in aquatic environment: the important role of natural biofilms. Science of the Total Environment.

[ref-18] Ighodaro OM, Akinloye OA (2018). First line defence antioxidants-superoxide dismutase (SOD), catalase (CAT) and glutathione peroxidase (GPX): their fundamental role in the entire antioxidant defence grid. Alexandria Journal of Medicine.

[ref-19] Jiang X, Guan X, Yao L, Zhang H, Jin X, Han Y (2016). Effects of single and joint subacute exposure of copper and cadmium on heat shock proteins in common carp (*Cyprinus carpio*). Biological Trace Element Research.

[ref-20] Justi LHZ, Silva JF, Santana MS, Laureano HA, Pereira ME, Oliveira CS, Guiloski IC (2025). Non-steroidal anti-inflammatory drugs and oxidative stress biomarkers in fish: a meta-analytic review. Toxicology Reports.

[ref-21] Kim J, Kang J (2017). Toxic effects on bioaccumulation and hematological parameters of juvenile rockfish *Sebastes schlegelii* exposed to dietary lead (Pb) and ascorbic acid. Chemosphere.

[ref-22] Kim J, Rhee J, Lee J, Dahms H, Lee J, Han K, Lee J (2010). Effect of cadmium exposure on expression of antioxidant gene transcripts in the river pufferfish, *Takifugu obscurus* (Tetraodontiformes). Comparative Biochemistry and Physiology Part C: Toxicology & Pharmacology.

[ref-23] Le Croizier G, Lacroix C, Artigaud S, Le Floch S, Raffray J, Penicaud V, Coquillé V, Autier J, Rouget M, Le Bayon N, Laë R, Tito De Morais L (2018). Significance of metallothioneins in differential cadmium accumulation kinetics between two marine fish species. Environmental Pollution.

[ref-24] Lee D, Choi YJ, Kim J (2022). Toxic effects of waterborne cadmium exposure on hematological parameters, oxidative stress, neurotoxicity, and heat shock protein 70 in juvenile olive flounder, *Paralichthys olivaceus*. Fish & Shellfish Immunology.

[ref-25] Lee J, Jo A, Lee D, Choi CY, Kang J, Kim J (2023). Review of cadmium toxicity effects on fish: oxidative stress and immune responses. Environmental Research.

[ref-26] Li E, Chen L, Zeng C, Yu N, Xiong Z, Chen X, Qin JG (2008). Comparison of digestive and antioxidant enzymes activities, haemolymph oxyhemocyanin contents and hepatopancreas histology of white shrimp, *Litopenaeus vannamei*, at various salinities. Aquaculture.

[ref-27] Li Y, Huang Y, He B, Liu R, Qu G, Yin Y, Shi J, Hu L, Jiang G (2020). Cadmium-binding proteins in human blood plasma. Ecotoxicology and Environmental Safety.

[ref-28] Li M, Zhu X, Tian J, Liu M, Wang G (2019). Dietary flavonoids from *Allium mongolicum* Regel promotes growth, improves immune, antioxidant status, immune-related signaling molecules and disease resistance in juvenile northern snakehead fish *(Channa argus*). Aquaculture.

[ref-29] Lin S, Bai Y, Xiong J (2022). Effects of dietary selenium on growth performance and blood parameters of juvenile Yellow River carp. Hubei Agricultural Sciences.

[ref-30] Liu Y, Chen Q, Li Y, Bi L, Jin L, Peng R (2022). Toxic effects of cadmium on fish. Toxics.

[ref-31] Liu Y, Wang J, Wei Y, Zhang H, Xu M, Dai J (2008). Induction of time-dependent oxidative stress and related transcriptional effects of perfluorododecanoic acid in zebrafish liver. Aquatic Toxicology.

[ref-32] Livak KJ, Schmittgen TD (2001). Analysis of relative gene expression data using real-time quantitative PCR and the 2^−ΔΔCT^ method. Methods.

[ref-33] Luo W, Wang D, Xu Z, Liao G, Chen D, Huang X, Wang Y, Yang S, Zhao L, Huang H, Li Y, Wei W, Long Y, Du Z (2020). Effects of cadmium pollution on the safety of rice and fish in a rice-fish coculture system. Environment International.

[ref-34] Mielcarek K, Nowakowski P, Puścion-Jakubik A, Gromkowska-Kępka KJ, Soroczyńska J, Markiewicz-Zukowska R, Naliwajko SK, Grabia M, Bielecka J, Zmudzińska A, Moskwa J, Karpińska E, Socha K (2022). Arsenic, cadmium, lead and mercury content and health risk assessment of consuming freshwater fish with elements of chemometric analysis. Food Chemistry.

[ref-35] Naz S, Hussain R, Ullah Q, Chatha AMM, Shaheen A, Khan RU (2021). Toxic effect of some heavy metals on hematology and histopathology of major carp (*Catla catla*). Environmental Science and Pollution Research.

[ref-36] Oršolić N, Jembrek MJ (2025). Targeting oxidative stress for disease. International of Journal of Molecular Sciences.

[ref-37] Othmani A, Magdouli S, Senthil Kumar P, Kapoor A, Chellam PV, Gökkuş Ö (2022). Agricultural waste materials for adsorptive removal of phenols, chromium (VI) and cadmium (II) from wastewater: a review. Environmental Research.

[ref-38] Paul JS, Small BC (2021). Chronic exposure to environmental cadmium affects growth and survival, cellular stress, and glucose metabolism in juvenile channel catfish (*Ictalurus punctatus*). Aquatic Toxicology.

[ref-39] Pei J, Pan X, Wei G, Hua Y (2023). Research progress of glutathione peroxidase family (GPX) in redoxidation. Frontiers in Pharmacology.

[ref-40] Perra M, Manca ML (2025). Recent trends in nanoantioxidants. Antioxidants.

[ref-41] Ray PD, Huang B, Tsuji Y (2012). Reactive oxygen species (ROS) homeostasis and redox regulation in cellular signaling. Cellular Signalling.

[ref-42] Salaah SM, Taha A, Medhat F, El-Naggar MM (2025). Physiological responses and histological alterations induced by pollution in the *Nile tilapia* from the Rosetta branch of the River Nile, Egypt. Scientific Reports.

[ref-43] Satarug S (2018). Dietary cadmium intake and its effects on kidneys. Toxics.

[ref-44] Sun J, Wang S, Yirong C, Wang S, Li S (2020). Cadmium exposure induces apoptosis, inflammation and immunosuppression through CYPs activation and antioxidant dysfunction in common carp neutrophils. Fish & Shellfish Immunology.

[ref-45] Sun M, Jing Y, Zhang T, Hu F, Chen Q, Liu G (2024). Effect of salinity on the toxicokinetics, oxidative stress, and metallothionein gene expression in *Meretrix meretrix* exposed to cadmium. Comparative Biochemistry and Physiology Part C: Toxicology & Pharmacology.

[ref-46] Wang H, Feng Y, Ming M, Song J, Chen Z, Xiao Z (2022). Amelioration of Cd-induced bioaccumulation, hematological parameters, and heat shock protein-related genes by Vitamin C on common carp. Comparative Biochemistry and Physiology Part C: Toxicology & Pharmacology.

[ref-47] Wang N, Gao C, Zhang P, Guan L, Wang Y, Qin Y, Li Y (2019). Effect of *Bacillus cereus* against cadmium induced hematological disturbances and immunosuppression in *Carassius auratus* gibelio. Fish & Shellfish Immunology.

[ref-48] Wang N, Guo Z, Zhang Y, Zhang P, Liu J, Cheng Y, Zhang L, Li Y (2020). Effect on intestinal microbiota, bioaccumulation, and oxidative stress of *Carassius auratu* s gibelio under waterborne cadmium exposure. Fish Physiology and Biochemistry.

[ref-49] Wei D, Huang T, Yin J, Zhang X (2022). Accumulation and distribution of cadmium in *Carassius auratus* and effect of water velocity on its scavenging. Journal of Huazhong Agricultural University.

[ref-50] Won TJ, Yu YB, Kang JH, Kim JH, Kang JC (2023). Effects on bioaccumulation, growth performance, hematological parameters, plasma components, and antioxidant responses in starry flounder *(Platichthys stellatu*s) exposed to dietary cadmium and ascorbic acid. Antioxidants.

[ref-51] Xu Y, Xiong H, Wang Z (2006). Effect of cadmium on liver catalase activity of *Carassius auratus*. Journal of Yangzhou University (Natural Science Edition).

[ref-52] Yang L, Fang Z, Zheng W, Wu Y, Ma G (2003). Experiment with effect of cadmium on activity of superoxide dismutase in gill and liver tissue of Crucian. Journal of Safety and Environment.

[ref-53] Yu Y, Choi J, Choi CY, Kang J, Kim J (2023). Toxic effects of microplastic (polyethylene) exposure: Bioaccumulation, hematological parameters and antioxidant responses in crucian carp, *Carassius carassius*. Chemosphere.

[ref-54] Zhang Y (2018). Cell toxicity mechanism and biomarker. Clinical and Translational Medicine.

[ref-55] Zhao X, Zhao W, Xu F, Shen Y, Bao Y, Yang B, Zhu T, Duan X, Jiao L, Monroig O, Zhou Q, Jin M (2024). Toxicity and detoxication assessment of juvenile black seabream (*Acanthopagrus schlegelii*) in response to dietary cadmium exposure: based on growth performance and stress indicators. Aquaculture Reports.

