# Supplementary figures and images for: Effects of waterborne cadmium exposure on hematological parameters, oxidative stress, and stress-related genes in crucian carp (Carassius auratus)
Source: PeerJ. 2026 Jul 8;14:e21528. doi: 10.7717/peerj.21528 (PMC13355607; doi:10.7717/peerj.21528)

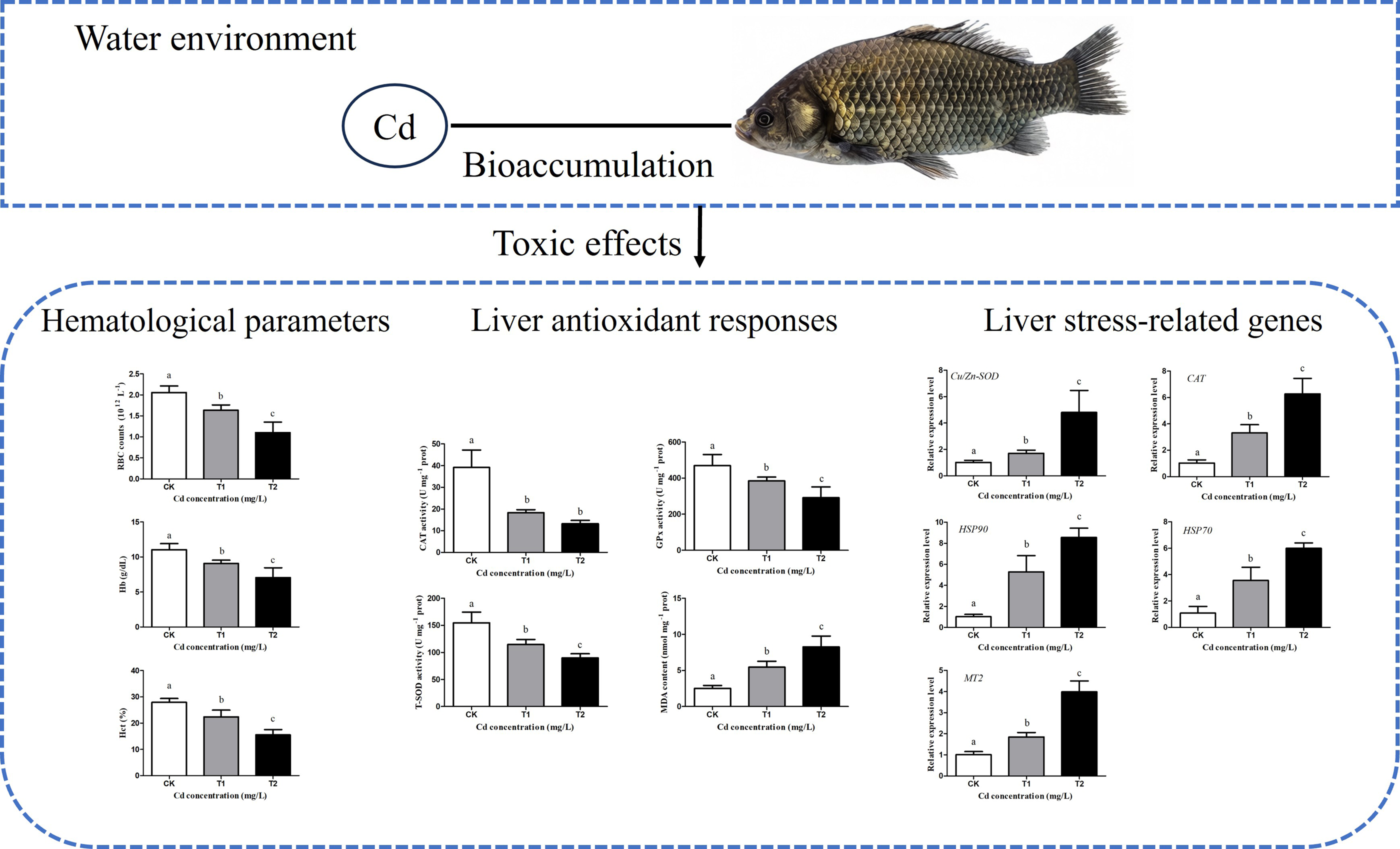

Supplement: Supplemental Information 1 — Cadmium exposure adversely impacts hematological parameters, induces oxidative stress, and results in significant alterations in the expression levels of stress-related genes. [file peerj-14-21528-s001.png]
